# Changes in Periodic Limb Movements of Sleep After the Use of Continuous Positive Airway Pressure Therapy: A Meta-Analysis

**DOI:** 10.3389/fneur.2022.817009

**Published:** 2022-06-02

**Authors:** Tzu-Chao Lin, Bing-Yan Zeng, Meng-Ni Wu, Tien-Yu Chen, Yen-Wen Chen, Pin-Yang Yeh, Ping-Tao Tseng, Chung-Yao Hsu

**Affiliations:** ^1^Department of Neurology, Kaohsiung Medical University Hospital, Kaohsiung, Taiwan; ^2^Department of Internal Medicine, E-DA Dachang Hospital, Kaohsiung, Taiwan; ^3^Department of Neurology and Sleep Disorders Center, Kaohsiung Medical University Hospital, Kaohsiung Medical University, Kaohsiung, Taiwan; ^4^Department of Neurology, Faculty of Medicine, College of Medicine, Kaohsiung Medical University, Kaohsiung, Taiwan; ^5^Department of Psychiatry, Tri-Service General Hospital, School of Medicine, National Defense Medical Center, Taipei, Taiwan; ^6^Institute of Brain Science, National Yang Ming Chiao Tung University, Taipei, Taiwan; ^7^Prospect Clinic for Otorhinolaryngology & Neurology, Kaohsiung, Taiwan; ^8^Department of Psychology, College of Medical and Health Science, Asia University, Taichung, Taiwan; ^9^Clinical Psychology Center, Asia University Hospital, Taichung, Taiwan; ^10^Institute of Biomedical Sciences, National Sun Yat-sen University, Kaohsiung, Taiwan

**Keywords:** continuous positive airway pressure (CPAP), periodic limb movement during sleep, obstructive sleep apnea, sleep medicine, respiration

## Abstract

**Background:**

Both obstructive sleep apnea (OSA) and periodic limb movements of sleep (PLMS) are common in the sleep laboratory. The severity of OSA can be improved by using continuous positive airway pressure (CPAP). However, increasing evidence has shown an elevated periodic limb movement index (PLMI) in patients with OSA who use CPAP, although the pathophysiology is still unknown. This meta-analysis aimed to investigate changes in PLMS after using CPAP and the potential pathophysiology of these changes.

**Methods:**

Clinical trials in adult humans investigating the comorbidity between PLMS and CPAP were identified and analyzed using random-effects model meta-analysis.

**Results:**

This meta-analysis included 14 studies comprising 2,938 patients with OSA. The PLMI was significantly increased after using CPAP with a difference in means of 1.894 (95% confidence interval = 0.651–3.138, *p* = 0.003). Subgroup analysis showed that CPAP was only significantly associated with an increase in PLMI in the patients without PLMS at baseline (*p* = 0.045) and in those with a baseline body-mass index <30 kg/m^2^ (*p* = 0.045). The use of CPAP, apnea-hypopnea index, and arousal index were positively correlated with changes in PLMI.

**Conclusion:**

These characteristics may serve as qualitative predictive indicators of changes in PLMI after CPAP usage. Further analysis of the quantitative relationships between PLMI and the predictive indicators may be warranted.

**Trial Registration:**

PROSPERO (registration number: CRD42021252635).

## Introduction

Obstructive sleep apnea (OSA) and periodic limb movements during sleep (PLMS) are commonly encountered in the sleep laboratory. The prevalence of OSA in the general population is about 2%−7% in adults (1), while that of PLMS is about 5%−11% (2). A high proportion of subjects with OSA have been reported to have concurrent PLMS, with a comorbidity rate of about 24%−28% (3). Continuous positive airway pressure (CPAP) is a standard treatment for OSA (4), and it has been shown to reduce the apnea–hypopnea index (AHI) and improve sleep quality (5).

Several studies have reported changes in the severity of PLMS during CPAP treatment in OSA patients (6). This phenomenon has also been reported in later clinical trials, however the results of these clinical trials have been inconsistent. Some trials have shown the potential beneficial effect of CPAP in OSA patients to reduce the severity of PLMS (7–9). For example, Kerkhofs and colleague found that CPAP could reduce the leg movement index with regards to time in bed [LMI (TIB)] from 391 per hour to 22.5 per hour in patients with OSA (8). In contrast, several recent trials have reported different findings (10). Wu et al. reported increased periodic limb movement index (PLMI) in OSA patients after using CPAP (10). However, another trial suggested that there might not be an association between the usage of CPAP in OSA patients and changes in PLMI (11).

Patients with a higher frequency of PLMS have been associated with more severe cardiovascular disease (12). In addition, the use of CPAP has been associated with a higher PLMI in certain populations, for example, the elderly, patients with chronic kidney disease, and those with higher baseline AHI (10, 13). This may imply the potential cardiovascular effect of residual PLMS after using CPAP, since CPAP *per se* aims to decrease the cardiovascular impact. Although the Sleep Apnea Cardiovascular Endpoints (SAVE) study reported that CPAP did not prevent cardiovascular events in patients with moderate-to-severe OSA (14), other study reported that CPAP reduced the risk of cardiovascular disease in patients with OSA and heart failure (15). Consequently, it is unclear which population may benefit from the cardiovascular protective effects of CPAP. Data on quantized, efficient and reliable indices for predicting an increase in the frequency of PLMS in OSA patients using CPAP are currently lacking. However, this is an important issue, as CPAP treatment may not provide as much cardiovascular protection in some patients as in others, and it could potentially lead to harm.

Patients with a higher PLMS frequency have also been reported to have worse sleep quality (16). Consequently, patients who experience an increase in the frequency of PLMS while using CPAP may also have a deterioration in the quality of sleep, which may in turn lower CPAP compliance. Identifying these patients before initiating CPAP could allow clinical physicians to inform the patient of the potential deterioration in sleep quality, thereby maintaining rapport and increasing the likelihood of better compliance with future medical plans. Therefore, it is vital to elucidate whether the application of CPAP affects the PLMI in patients with OSA.

The aim of this meta-analysis was to clarify the changes in PLMI associated with the use of CPAP in OSA patients, and to identify potential characteristics of OSA patients to predict these changes in PLMI.

## Methods and Materials

**Figure 1:**
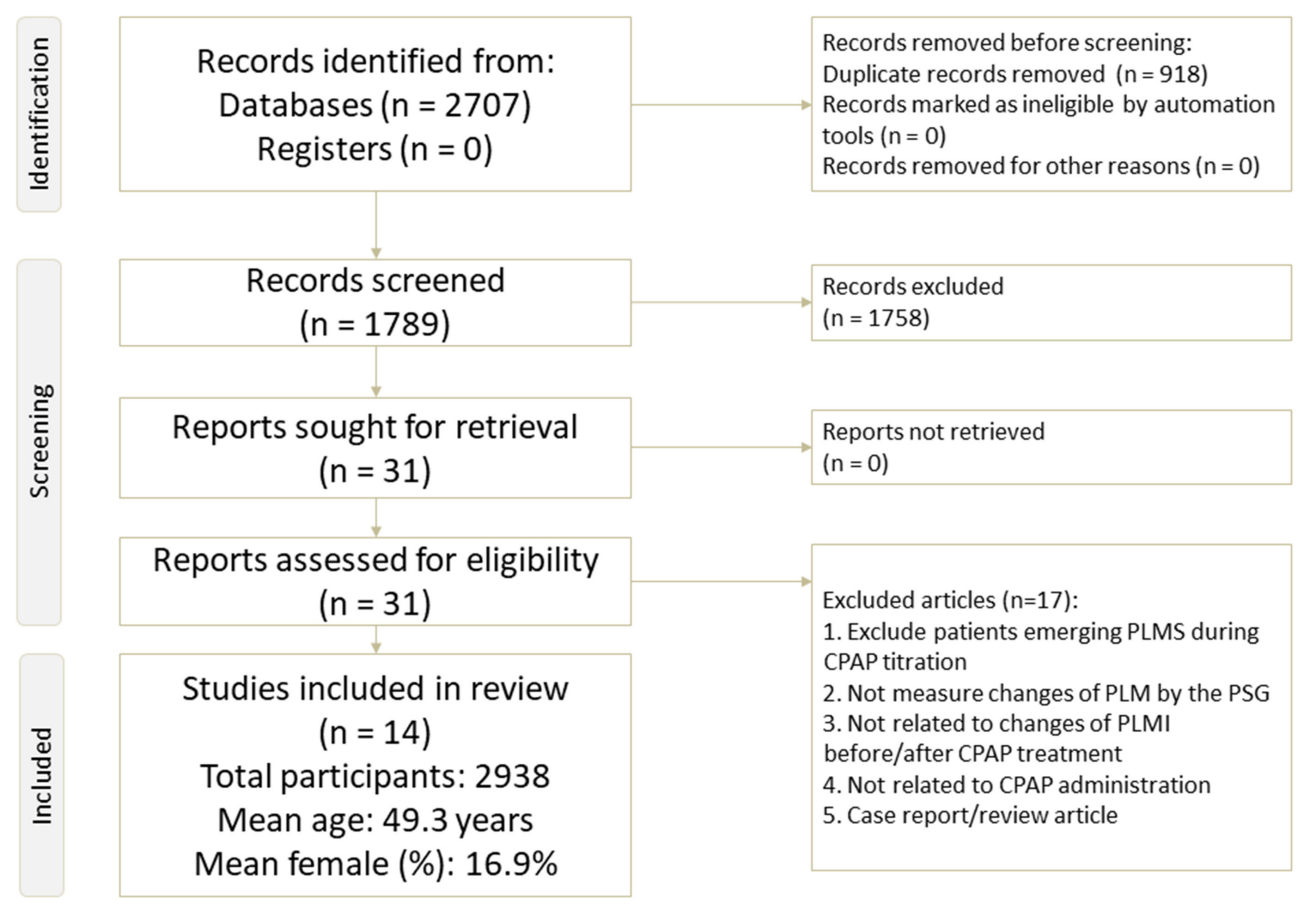
Flowchart of the current meta-analysis.

In this study, we followed the *Preferred Reporting Items for Systematic Reviews and Meta-Analyses* (PRISMA) 2020 guidelines (17) (Supplementary Table 1 and Figure 1) and AMSTAR2 (a measurement tool to assess systematic reviews) guidelines (18). The current study is registered in PROSPERO (registration number: CRD42021252635). In addition, the study protocol of the current meta-analysis (Appendix: study protocol) was approved by the Institutional Review Board of Tri-Service General Hospital (TSGHIRB: B-109-29).

### Database Searches and Identification of Eligible Papers

Two well-trained and independent authors (T-CL and B-YZ) performed electronic searches of PubMed, Embase, ScienceDirect, ClinicalKey, Cochrane Library, ProQuest, Web of Science, and ClinicalTrials.gov using the keywords (CPAP OR continuous positive airway pressure) AND (PLM OR periodic limb movement during sleep OR PLMD OR periodic limb movement disorder) up to May 8th, 2021 (detailed keyword and search results are shown in Supplementary Table 2). In the initial stage, both authors screened the titles and abstracts for eligibility through consensus. If a consensus could not be reached, a third author (P-TT) was consulted. Furthermore, in order to increase the number of included studies, we hand searched the reference lists of review articles and clinical guidelines (9).

### Inclusion and Exclusion Criteria

The PICO applied in this meta-analysis was: (1) Patient or Problem: patients with sleep apnea, either central or obstructive; (2) Intervention: CPAP; (3) Comparator: before and after CPAP treatment; and (4) Outcome: changes in PLMI.

Therefore, the inclusion criteria when screening the eligible studies identified from the search were: (1) formal published articles investigating changes in PLMI before and after CPAP treatment, either in forms of a pre-post comparison design or placebo-controlled trial; and (2) articles that were clinical trials in adult humans. To expand the potential eligibility, we did not set any limitations. The exclusion criteria were: (1) animal studies; and (2) articles not related to changes in PLMI before and after CPAP treatment.

### Methodological Quality Appraisal

To investigate the methodological quality of the recruited studies, we used the Jadad scale, which ranges from zero (poor quality) to five (high quality) (19).

### Primary Outcome

The primary outcome was the change in PLMI, which was defined as the rate of PLM during sleep (PLM/hour) recorded by polysomnography based on either the American Association of Sleep Medicine (AASM) guidelines (20) or Coleman's criteria (21).

### Secondary Outcome

The secondary outcome was defined as the change in PLM with arousal index (PLMAI) recorded by polysomnography based on either the AASM guidelines (20) or Coleman's criteria (21).

### Data Extraction and Management

Based on the predetermined clinical variables of interest, the two authors (T-CL and B-YZ) extracted data from the recruited studies, including mean age (years), gender, body mass index (BMI), sleep parameters recorded by polysomnography, including total sleep time (TST) (min), sleep efficiency (%), percentage of deep sleep phase (i.e. non-rapid eye movement (NREM) stage, N3 sleep phase) (%), percentage of rapid eye movement (REM) sleep phase (%), REM sleep onset latency (min), sleep onset latency (min), AHI, arousal index (AI), oxygen desaturation index (ODI), mean O_2_ saturation, Epworth Sleepiness Scale (ESS), and Jadad scores.

In situations where data were unavailable in a study, we tried to contact the corresponding authors to request the original data on at least two different occasions.

### Meta-Analysis

Based on the presumed heterogeneity of the populations among the recruited studies, the current meta-analysis was conducted using random-effects meta-analysis models. The meta-analysis procedure was performed using Comprehensive Meta-Analysis software, version 3 (Biostat, Englewood, NJ). We chose differences in means and 95% confidence intervals (95% CIs) as the main effect sizes (ESs) of the primary outcome (change in PLMI) and secondary outcome (change in PLMAI). Two-tailed *p* values < 0.05 were considered to be statistically significant.

### Heterogeneity, Publication Bias, and Sensitivity Test

To detect potential heterogeneity, we used the *Q* statistic and corresponding *p* values (22). In addition, we visually inspected funnel plots when there were fewer than 10 datasets (23), and used Egger's regression test when there were 10 or more datasets (24). We used the Duval and Tweedie trim and fill test when there was evidence of publication bias (25).

Sensitivity testing was done using the one study removal method, in which one study was excluded from the analysis at a time, to observe whether the significant or insignificant results of the meta-analysis were biased by an outlier (26).

### Meta-Regression and Subgroup Meta-Analysis

We used meta-regression and subgroup meta-analyses to identify potential sources of heterogeneity and confounding factors. Specifically, in situations of at least 10 datasets, we performed meta-regression using the unrestricted maximum likelihood method. The variables of interest for meta-regression were mean age, proportion of females, BMI, TST, sleep efficiency, percentage of deep sleep phase, percentage of REM sleep phase, REM sleep onset latency, sleep onset latency, AHI, AI, ODI, mean O_2_ saturation, ESS, and Jadad scores. In addition, we performed subgroup meta-analysis when there were at least three datasets (27). We also performed subgroup meta-analysis among different groups with/without baseline PLMS, and BMI higher/lower than 30 kg/m^2^.

## Results

### Study Selection

The protocol of study selection for the current meta-analysis is depicted in Figure 1. In brief, 29 full-text articles were considered to be eligible for screening. Among them, 15 were excluded because they did not fulfill the inclusion criteria (reasons summarized in Figure 1 and Supplementary Table 3), and the remaining 14 articles were enrolled in this meta-analysis (3, 7, 8, 10, 13, 28–36) (Table 1).

**Table 1:** Characteristics of the included studies.

**References**	**Baseline PLMS**	**Subjects**	**Mean age**	**Female (%)**	**Mean BMI**	**Baseline AHI**	**Country**
Budhiraja et al. (35)	Partial yes	558	51.6 ± 12.0	34.6	32.2	40.1	USA
Murase et al. (34)	Partial yes	60	58.0 ± 10.0	15	28.0	39.4	Japan
Wu et al. (32)	No	60	46.2 ± 12.8	86.7	28.2	49.4	Taiwan
Yang et al. (36) PLM persist	Yes	32	51.4 ± 10.0	28.1	27.3	52.7	China
Yang et al. (36) CPAP disappear PLM	Yes	62	46.7 ± 11.9	8.1	28.0	61.2	
Yang et al. (36) CPAP emergent PLM	No	85	50.3 ± 10.8	7.1	26.8	62.4	
Yang et al. (36) non PLM at all	No	649	44.7 ± 10.3	8.0	27.6	58.2	
Mwenge et al. (33)	Partial yes	160	57.7 ± 12.0	13.8	32.7	53.0	Belgium
Aritake-Okada et al. (13) CPAP disappear PLM	Yes	40	55.7 ± 15.2	17.5	28.3	42.8	Japan
Aritake-Okada et al. (13) CPAP emergent PLM	No	80	51.6 ± 13.7	7.5	28.6	55.1	
Aritake-Okada et al. (13) non PLM at all	No	810	46.3 ± 12.0	6.2	28.1	46.0	
Aritake-Okada et al. (13) PLM persist	Yes	67	56.6 ± 11.9	16.4	26.9	44.5	
Hedli et al. (31)	Partial yes	39	61.7	30.8	30.9	38.2	USA
Benz et al. (10)	Partial yes	16	65.2 ± 14.2	37.5	NA	49.2	USA
Drigo et al. (7) 5 < PLMI <25	Yes	28				32.3	Italy
Drigo et al. (7) PLMI <5	No	28	54.3 ± 10.8	21.5	30.3	26.0	
Drigo et al. (7) PLMI > 25	Yes	9				32.2	
Baran et al. (3)	Yes	86	53.8 ± 11.8	40	34.41	38.74	USA
Noseda et al. (8)	Yes	14	54.0 ± 12.0	7.1	29.6	26.1	Belgium
Carelli et al. (30)	Partial yes	26	50.9 ± 9.6	11.5	32.94	NA	France
Yamashiro and Kryger (28)	Yes	15	52.7 ± 8.5	20	NA	30.5	Canada
Fry et al. (29)	Yes	14	54.0 ± 11.0	9.1	NA	NA	USA

*AHI, apnea-hypopnea index; BMI, body mass index; CPAP, continuous positive airway pressure; NA, not available; PLMS, periodic limb movements of sleep; PLMAI, periodic limb movement with arousal index; PLMI, periodic limb movement index*.

Among the 14 articles, 13 were pre-post comparisons and only one was a randomized controlled trial. All of these articles provided data on changes in PLMI before and after CPAP treatment (3, 7, 8, 10, 13, 28–36) (participants = 2,938, mean age = 49.3 years, mean female proportion = 16.9%). Furthermore, five provided changes in PLMI in patients without baseline PLMS or baseline PLMI <5 (7, 13, 32, 33, 36) (participants = 1,872, mean age = 47.2 years, mean female proportion = 10.4%), and seven provided changes in PLMI in those with baseline PLMS or baseline PLMI ≥5 (3, 7, 8, 13, 28, 29, 36) (participants = 367, mean age = 53.1 years, mean female proportion = 21.7%). In addition, five articles provided changes in PLMAI before and after CPAP treatment (7, 10, 28, 29, 31) (participants = 149, mean age = 57.2 years, mean female proportion = 24.3%). The patients in the recruited studies had a diagnosis of sleep-disordered breathing plus PLMS (28), sleep-disordered breathing plus chronic kidney disease (10), sleep-disordered breathing only (7, 31, 34, 35), sleep apnea-hypopnea syndrome with high leg activity (8), or obstructive sleep apnea syndrome only (3, 13, 29, 30, 36).

### Methodological Quality of the Included Studies

Across the 14 studies, the average Jadad score was 0.8 with a standard deviation (SD) of 0.4 (Supplementary Table 4).

### Primary Outcome: Changes in PLMI Before and After CPAP Treatment

**Figure 2:**
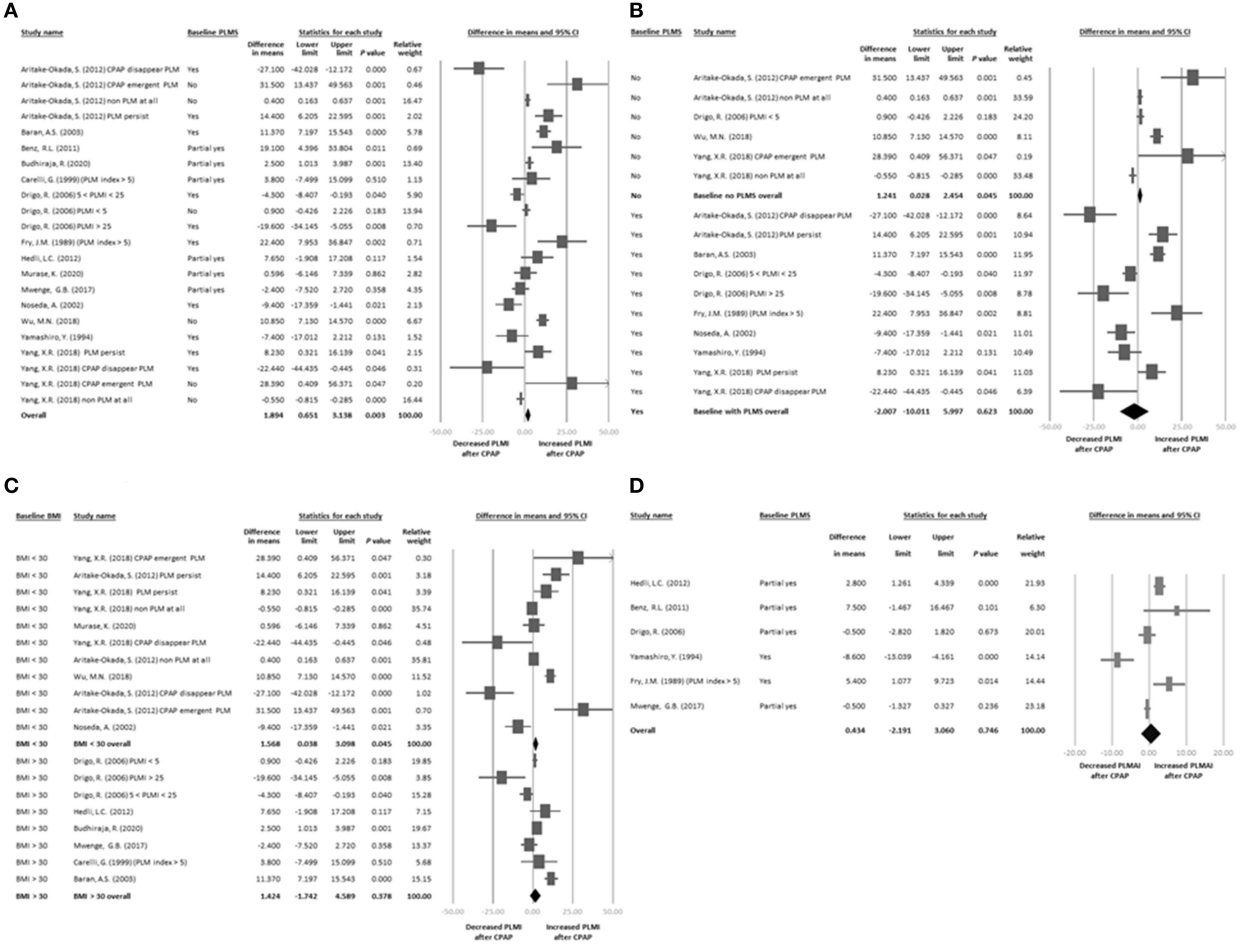
**(A)** Forest plot of changes in PLMI before and after CPAP treatment. **(B)** Forest plot of changes in PLMI before and after CPAP treatment: subgroup baseline PLMS. **(C)** Forest plot of changes in PLMI before and after CPAP treatment: subgroup baseline BMI. **(D)** Forest plot of changes in PLMAI before and after CPAP treatment. In **(A–D)**, effect sizes >0 indicated that CPAP treatment was associated with increased PLMI or PLMAI. AHI, apnea-hypopnea index; BMI, body mass index; CI, confidence interval; CPAP, continuous positive airway pressure; PLMS, periodic limb movements of sleep; PLMAI, periodic limb movement with arousal index; PLMI, periodic limb movement index; PSG, polysomnography.

The 14 articles provided 22 datasets of changes in PLMI before and after CPAP treatment (3, 7, 8, 10, 13, 28–36). The main results of the meta-analysis revealed that PLMI was significantly increased after CPAP treatment with a difference in means of 1.894 (95% CI = 0.651–3.138, *p* = 0.003; Figure 2A) with significant heterogeneity (*Q* value = 187.278, df = 21, *p* < 0.001; *I*^2^ = 88.787%) but not publication bias *via* Egger's regression (*t*-value = 1.390, df = 20, *p* = 0.180).

### Sensitivity Test

The sensitivity test via one study removed revealed that the main results of the meta-analysis became insignificant after removing the dataset by Wu et al. (32) (difference in means = 1.193, 95% CI = −0.009 to 2.396, p = 0.052).

### Meta-Regression

The meta-regression showed that there were only significant positive associations between changes in PLMI and female proportion (*k* = 22, slope = 0.155, *p* < 0.001), AHI at baseline (*k* = 20, slope = 0.157, *p* = 0.048), and AI at baseline (*k* = 10, slope = 1.744, *p* = 0.002), but not mean age (*p* = 0.556) or BMI (*p* = 0.194) (Supplementary Figures 1A–E).

### Subgroup Meta-Analysis: The Presence of Baseline PLMS

We then performed subgroup meta-analysis according to the baseline PLMI status among the recruited patients. According to the International Classification of Sleep Disorders (ICSD-3), a PLMI of 5 or more is regarded as abnormal (14). The four articles that provided changes in PLMI in patients without baseline PLMS or baseline PLMI <5 included a total of six datasets (7, 13, 32, 36). The subgroup meta-analysis results revealed a significant increase in PLMI after CPAP treatment (*k* = 6, difference in means = 1.241, 95% CI = 0.028–2.454, *p* = 0.045; Figure 2B). In addition, seven studies with 10 datasets (3, 7, 8, 13, 28, 29, 36) provided changes in PLMI in patients with baseline PLMS ≥5. The subgroup meta-analysis results revealed that there was no significant change in PLMI after CPAP treatment (*k* = 10, difference in means = −2.007, 95% CI = −10.011 to 5.997, *p* = 0.623; Figure 2B).

### Subgroup Meta-Analysis: Differences in Baseline BMI

We performed further subgroup meta-analysis based on a cut-off baseline BMI of 30 kg/m^2^. Five studies with seven datasets (8, 13, 32, 34, 36) provided data on patients with a baseline BMI <30 kg/m^2^. The subgroup meta-analysis results revealed a significant increase in PLMI after CPAP treatment (*k* = 11, difference in means = 1.568, 95% CI = 0.038–3.098, *p* = 0.045; Figure 2C). In addition, six studies with eight datasets (3, 7, 30, 31, 33, 35) provided data on patients with a baseline BMI ≥30 kg/m^2^. The subgroup meta-analysis results revealed that there was no significant change in PLMI after CPAP treatment (*k* = 8, difference in means = 1.424, 95% CI = −1.742 to 4.589, *p* = 0.378; Figure 2C).

### Secondary Outcome: Changes in PLMAI Before and After CPAP Treatment

Six of the articles provided six datasets of changes in PLMAI before and after CPAP treatment (7, 10, 28, 29, 31, 33). The main results of the meta-analysis revealed that there was no significant change in PLMAI before or after CPAP treatment (*k* = 6, difference in means = 0.434, 95% CI = −2.191 to 3.060, *p* = 0.746; Figure 2D) with significant heterogeneity (*Q* value = 37.238, df = 5, *p* < 0.001; *I*^2^ = 86.573%) and significant publication bias *via* inspection of funnel plots (Supplementary Figure 2). The adjusted estimated ESs by Duval and Tweedie's trim and fill test remained insignificant (adjusted difference in means = −0.045, 95% CI = −2.629 to 2.539).

### Sensitivity Test

The sensitivity test *via* one study removed revealed that the insignificant results of the meta-analysis did not change by removing of any one of the studies. Therefore, the insignificant results were not due to an outlier.

## Discussion

To the best of our knowledge, this is the first meta-analysis to address the changes in PLMI associated with CPAP treatment. The major finding of the current study is the significant increase in PLMI after using CPAP, which was independent of the presence of PLMS at baseline and a baseline BMI <30 kg/m^2^. Specifically, the PLMI was significantly increased after CPAP treatment with strong positive associations with baseline AHI and AI (slope = 0.157, *p* = 0.048 and slope = 1.744, *p* = 0.002, respectively), while there were no significant changes in PLMAI. Furthermore, only the subgroups with a baseline minimal PLMI (<5 or without baseline PLMS) and BMI <30 kg/m^2^ had a significant increase in PLMI associated with CPAP treatment.

The increase in PLMI after CPAP treatment found in this meta-analysis is consistent with previous studies (7, 13). However, clear pathophysiological explanations for the effect of CPAP on the increase in PLMI are still lacking. Baran et al. (3) hypothesized that CPAP may increase PLMI either through OSA-related sleep fragmentation or CPAP-triggered sleep fragmentation and bodily discomfort. However, further studies are needed to elucidate this issue.

Our subgroup meta-analysis showed that the OSA patients without PLMS (or PLMI <5) at baseline had an increase in PLMI after CPAP treatment, but those with PLMS (or PLMI ≥5) at baseline showed no obvious change in PLMI. Pathophysiological explanations for this result are also lacking. In a recent study focusing on OSA patients who received CPAP treatment, emerging PLMS (increase in PLMS by 5/h with the disappearance of respiratory events after CPAP treatment) accounted for about 30% of the patients, which inevitably increased PLMI scores after CPAP treatment (16). This may explain the elevation in PLMI after CPAP treatment in the subgroup with PLMI <5. In the same study, the authors suggested that serum ferritin could predict an increase in PLMI rather than the initial PLMI score. Therefore, a low ferritin level, rather than initial low PLMI, may be a predictor of a higher frequency of PLMS after CPAP treatment. Accordingly, despite the subgroup results, ferritin level may be more pathophysiologically useful than baseline PLMI in predicting changes in PLMI after CPAP treatment. However, this hypothesis requires further verification as it was based on only a single study, and because ferritin is not more pathologically plausible than baseline PLMI in predicting an increase in PLMI.

Our results also showed that the patients with higher baseline AHI had a greater increase in PLMI after using CPAP. Explanations for this finding are lacking. Some studies have reported that arousals showed the same tendency as PLMI in patients using CPAP (3, 29). Although some previous studies have reported a tendency of PLMI, arousals and AHI to increase in patients using CPAP, further studies are needed to investigate these effects before reasonable pathophysiological explanations can be drawn.

In our main results, BMI was not correlated with CPAP treatment. However, in subgroup analysis, those with a BMI <30 kg/m^2^ had an increase in PLMI after using CPAP. PLMS is known to be most active during the transition from wakefulness to sleep and most NREM sleep (light NREM sleep), and then to be attenuated during very deep NREM sleep and even more during REM sleep (16). According to a case control study, after 3 months of CPAP treatment, people with normal BMI (NG, range from 18 to 25 kg/m^2^) seemed to have an increase in NREM sleep (baseline 69.6% of TID increasing to 70.9%), whereas the obese group (OG, range from 30 to 40 kg/m^2^) and severely obese group (SOG, >40 kg/m^2^) had a decrease (OG baseline 72.9% TID decreasing to 67.8%, and SOG baseline 68.7% TID decreasing to 65.9%). Moreover, although REM sleep stage increased in all three groups, the NG group had the smallest increase (NG 4.3%, OG 9.2% and OSG 10.5%) and final total REM TIB percentage (NG 12.6%, OG 15.0% and OSG 15.2%) (37). However, CPAP treatment in obese patients may increase neural respiratory drive (38), which may increase the frequency of arousals (39), thus increasing wakefulness-sleep transition times and worsening PLMS. Therefore, PLMI may increase in non-obese patients due to an increase in the proportion of NREM sleep. Whereas in obese patients, although improvements in PLMS may decrease the proportion of NREM sleep and increase REM sleep, this may be offset by an increase in the frequency of neural-respiratory-drive-related arousals and wakefulness-sleep transition times. In summary, our main results showed that BMI was not significantly correlated with CPAP treatment. However, recent studies (16, 37–39) support our subgroup findings that an increase in PLMI was associated with CPAP treatment in patients with a BMI <30 kg/m^2^, but not in those with a BMI ≥30 kg/m^2^.

In conclusion, PLMI was significantly increased after CPAP treatment. An increase in PLMI could be predicted using factors including baseline high AHI, baseline high AI and BMI <30 kg/m^2^. Baseline PLMI <5 (or without baseline PLMS) was a possible pathophysiological predictor. Although ferritin level may be a more convincing predictor, further studies are needed to investigate this hypothesis. Patients with OSA and more predictive factors may have a higher risk of severe PLMS after CPAP treatment.

There are some limitations to the current study. First, the wide variety of heterogeneity among the included studies, such as differences in subject selection criteria (one of the enrolled studies used PLMI >5 on polysomnography with CPAP as their inclusion criteria and excluded patients without PLMS after CPAP), different ages, comorbidities of the enrolled patients, different scoring criteria of sleep disordered breathing and PLMS, different equipment to measure PLMS (i.e. traditional polysomnography and actigraphy), and different methods of titrating CPAP (i.e. manual titration and auto-titration). Second, night-to-night variability of PLMI and PLMAI in two consecutive nights occurred in 27 and 19% of PLMS, respectively (40), and it is unclear whether night-to-night variability may worsen or improve after long-term CPAP therapy. In this study, the interval between the first polysomnography for diagnosis and polysomnography with CPAP ranged from 1 day to 2–7 months. The night-to-night variability *per se* and the different intervals between the two polysomnography sessions may have affected comparisons of absolute changes in PLMI. Third, the PLMI may differ between different sleep stages and time of night. One study conducted standard polysomnography during the first half of the night and polysomnography with CPAP during the second half of the night, which may also have affected the results. Fourth, not all of the included studies confirmed PLM using the standard method (i.e. polysomnography), and some used actigraphy to measure PLM, which may not be sufficiently sensitive. Fifth, since there are two subtypes of sleep apnea (i.e. central sleep apnea and obstructive sleep apnea), it would be clinically helpful to conduct subgroup analysis based on these two subtypes. However, because few studies provided such detailed subtype information, further subgroup analysis could not be done. Sixth, because of the natural limitation of a meta-analysis, we could only observe the phenomenon of changes in PLMI related to CPAP treatment, but could not directly conclude the physiopathology responsible for these changes. Finally, and most importantly, every study except that by Budhiraja et al. (35) performed peri-CPAP treatment. It is possible that the increase in PLMI with CPAP treatment could be related to a circadian variation. In addition, the increase in PLMI could just be related to a tendency to be higher in the morning hours rather than the influence of CPAP. It should be noted that Budhiraja et al. (35) (a randomized controlled trial and also the third largest study in the meta-analysis) did not show any difference in the change in PLMI when comparing sham CPAP to active CPAP over a 6-month period.

## Conclusion

*Conclusion:* The main findings of this meta-analysis were that patients with OSA had a significant increase in PLMI after CPAP treatment, while there were no significant changes in PLMAI. AHI and AI were strongly and positively correlated with changes in PLMI. In addition, PLMI was also increased in the patients with PLMI <5 and BMI <30 kg/m^2^. The proposed predictors may serve as indicators to qualitatively predict an increase in PLMI, and they may serve as qualitative predictive indicators of changes in PLMI after CPAP usage.

*Future recommendation:* Future studies analyzing the quantitative relationship between PLMI and these indicators may be warranted.

## Author Contributions

T-CL, P-TT, and B-YZ performed manuscript drafting, data analysis, and visualization. M-NW, T-YC, Y-WC, and P-YY contributed to concept formation, data curation, literature review, study design, and manuscript revision. C-YH was responsible for collecting information from the other authors, manuscript major revision, software analysis, and manuscript submission. All authors contributed to the article and approved the submitted version.

## Conflict of Interest

The authors declare that the research was conducted in the absence of any commercial or financial relationships that could be construed as a potential conflict of interest.

## Publisher's Note

All claims expressed in this article are solely those of the authors and do not necessarily represent those of their affiliated organizations, or those of the publisher, the editors and the reviewers. Any product that may be evaluated in this article, or claim that may be made by its manufacturer, is not guaranteed or endorsed by the publisher.

## Abbreviations

AASM: American Association of Sleep Medicine
AHI: apnea-hypopnea index
AI: arousal index
BMI: body mass index
CI: confidence interval
CPAP: continuous positive airway pressure
ES: effect size
ESS: Epworth Sleepiness Scale
ODI: oxygen desaturation index
OSA: obstructive sleep apnea
PLMS: periodic limb movements of sleep
PLMAI: periodic limb movements with arousal index
PLMI: periodic limb movement index
PRISMA: Preferred Reporting Items for Systematic Reviews and Meta-Analyses
REM: rapid eye movement
TST: total sleep time.
