# Supplementary material for: Changes in Periodic Limb Movements of Sleep After the Use of Continuous Positive Airway Pressure Therapy: A Meta-Analysis
Source: Front Neurol. 2022 Jun 2;13:817009. doi: 10.3389/fneur.2022.817009 (PMC9202316; doi:10.3389/fneur.2022.817009)
Supplement: Supplementary file 1 [file Data_Sheet_1.pdf]

eTable 1: Checklist of PRISMA 2020 guideline

Section and Topic	Item #	Checklist item	Page where item is reported
**TITLE**			
Title	1	Identify the report as a systematic review.	1
**ABSTRACT**			
Abstract	2	See the PRISMA 2020 for Abstracts checklist.	3
**INTRODUCTION**			
Rationale	3	Describe the rationale for the review in the context of existing knowledge.	5-6
Objectives	4	Provide an explicit statement of the objective(s) or question(s) the review addresses.	5-6
**METHODS**			
Eligibility criteria	5	Specify the inclusion and exclusion criteria for the review and how studies were grouped for the syntheses.	7-8
Information sources	6	Specify all databases, registers, websites, organisations, reference lists and other sources searched or consulted to identify studies. Specify the date when each source was last searched or consulted.	7-8
Search strategy	7	Present the full search strategies for all databases, registers and websites, including any filters and limits used.	7-8
Selection process	8	Specify the methods used to decide whether a study met the inclusion criteria of the review, including how many reviewers screened each record and each report retrieved, whether they worked independently, and if applicable, details of automation tools used in the process.	7-8
Data collection process	9	Specify the methods used to collect data from reports, including how many reviewers collected data from each report, whether they worked independently, any processes for obtaining or confirming data from study investigators, and if applicable, details of automation tools used in the process.	7-8
Data items	10a	List and define all outcomes for which data were sought. Specify whether all results that were compatible with each outcome domain in each study were sought (e.g. for all measures, time points, analyses), and if not, the methods used to decide which results to collect.	8-9
	10b	List and define all other variables for which data were sought (e.g. participant and intervention characteristics, funding sources). Describe any assumptions made about any missing or unclear information.	8-9
Study risk of bias assessment	11	Specify the methods used to assess risk of bias in the included studies, including details of the tool(s) used, how many reviewers assessed each study and whether they worked independently, and if applicable, details of automation tools used in the process.	8-9
Effect measures	12	Specify for each outcome the effect measure(s) (e.g. risk ratio, mean difference) used in the synthesis or presentation of results.	8-9
Synthesis methods	13a	Describe the processes used to decide which studies were eligible for each synthesis (e.g. tabulating the study intervention characteristics and comparing against the planned groups for each synthesis (item #5)).	8-9
	13b	Describe any methods required to prepare the data for presentation or synthesis, such as handling of missing summary statistics, or data conversions.	9-10

Section and Topic	Item #	Checklist item	Page where item is reported
	13c	Describe any methods used to tabulate or visually display results of individual studies and syntheses.	9-10
	13d	Describe any methods used to synthesize results and provide a rationale for the choice(s). If meta-analysis was performed, describe the model(s), method(s) to identify the presence and extent of statistical heterogeneity, and software package(s) used.	9-10
	13e	Describe any methods used to explore possible causes of heterogeneity among study results (e.g. subgroup analysis, meta-regression).	9-10
	13f	Describe any sensitivity analyses conducted to assess robustness of the synthesized results.	9-10
Reporting bias assessment	14	Describe any methods used to assess risk of bias due to missing results in a synthesis (arising from reporting biases).	9-10
Certainty assessment	15	Describe any methods used to assess certainty (or confidence) in the body of evidence for an outcome.	9-10
**RESULTS**			
Study selection	16a	Describe the results of the search and selection process, from the number of records identified in the search to the number of studies included in the review, ideally using a flow diagram.	11-12 Fig 1, eTab 2
	16b	Cite studies that might appear to meet the inclusion criteria, but which were excluded, and explain why they were excluded.	11-12, eTab 3
Study characteristics	17	Cite each included study and present its characteristics.	11-12, Tab 1
Risk of bias in studies	18	Present assessments of risk of bias for each included study.	11-12
Results of individual studies	19	For all outcomes, present, for each study: (a) summary statistics for each group (where appropriate) and (b) an effect estimate and its precision (e.g. confidence/credible interval), ideally using structured tables or plots.	11-12, Tab 1
Results of syntheses	20a	For each synthesis, briefly summarise the characteristics and risk of bias among contributing studies.	12-13, Fig 2
	20b	Present results of all statistical syntheses conducted. If meta-analysis was done, present for each the summary estimate and its precision (e.g. confidence/credible interval) and measures of statistical heterogeneity. If comparing groups, describe the direction of the effect.	12-13, Fig 2
	20c	Present results of all investigations of possible causes of heterogeneity among study results.	12-13
	20d	Present results of all sensitivity analyses conducted to assess the robustness of the synthesized results.	12-14
Reporting biases	21	Present assessments of risk of bias due to missing results (arising from reporting biases) for each synthesis assessed.	13-14
Certainty of evidence	22	Present assessments of certainty (or confidence) in the body of evidence for each outcome assessed.	13-14
**DISCUSSION**			
Discussion	23a	Provide a general interpretation of the results in the context of other evidence.	15-17
	23b	Discuss any limitations of the evidence included in the review.	17
	23c	Discuss any limitations of the review processes used.	17
	23d	Discuss implications of the results for practice, policy, and future research.	17-18

Section and Topic	Item #	Checklist item	Page where item is reported
**OTHER INFORMATION**			
Registration and protocol	24a	Provide registration information for the review, including register name and registration number, or state that the review was not registered.	3
	24b	Indicate where the review protocol can be accessed, or state that a protocol was not prepared.	3
	24c	Describe and explain any amendments to information provided at registration or in the protocol.	3
Support	25	Describe sources of financial or non-financial support for the review, and the role of the funders or sponsors in the review.	21-22
Competing interests	26	Declare any competing interests of review authors.	21-22
Availability of data, code and other materials	27	Report which of the following are publicly available and where they can be found: template data collection forms; data extracted from included studies; data used for all analyses; analytic code; any other materials used in the review.	21-22

The current checklist followed the latest PRISMA 2020 guideline [1].

Reference

1. Page MJ, McKenzie JE, Bossuyt PM, Boutron I, Hoffmann TC, Mulrow CD, Shamseer L, Tetzlaff JM, Akl EA, Brennan SE, et al: The PRISMA 2020 statement: an updated guideline for reporting systematic reviews. BMJ. 2021; 372:n71.

eTable 2: Keywords applied in each database

Database	Keyword	Filter	Date	Result
PubMed	(CPAP OR continuous positive airway pressure) AND (PLM OR periodic limb movement during sleep OR PLMD OR periodic limb movement disorder)	NA	2021/5/8	83
Embase	(CPAP OR continuous positive airway pressure) AND (PLM OR periodic limb movement during sleep OR PLMD OR periodic limb movement disorder)	NA	2021/5/8	199
ScienceDirect	(CPAP OR continuous positive airway pressure) AND (PLM OR periodic limb movement during sleep OR PLMD OR periodic limb movement disorder)	research article	2021/5/8	675
ClinicalKey	(CPAP OR continuous positive airway pressure) AND (PLM OR periodic limb movement during sleep OR PLMD OR periodic limb movement disorder)	NA	2021/5/8	79
Cochrane CENTRAL	(CPAP OR continuous positive airway pressure) AND (PLM OR periodic limb movement during sleep OR PLMD OR periodic limb movement disorder)	NA	2021/5/8	14
ProQuest	(CPAP OR continuous positive airway pressure) AND (PLM OR periodic limb movement during sleep OR PLMD OR periodic limb movement disorder)	NA	2021/5/8	1590
Web of Science	(CPAP OR continuous positive airway pressure) AND (PLM OR periodic limb movement during sleep OR PLMD OR periodic limb movement disorder)	NA	2021/5/8	67
ClinicalTrials.gov	(CPAP OR continuous positive airway pressure) AND (PLM OR periodic limb movement during sleep OR PLMD OR periodic limb movement disorder)	NA	2021/5/8	0

Abbreviation: NA: not applied

eTable 3: Excluded studies and reason

Reason	Numbers	References
Case report	5	[1-5]
Exclude patients emerging PLMS during CPAP titration	1	[6]
Investigate Adaptive Servo-Ventilation but not CPAP	1	[7]
Not included adult patients	1	[8]
Not measure changes of PLM by the PSG	1	[9]
Not related to changes of PLMI before/after CPAP treatment	4	[10-14]
Not related to CPAP administration	2	[15, 16]
Review article	1	[17]

Abbreviation: CPAP: continuous positive airway pressure; PLMS: periodic limb movements of sleep; PLMI: periodic limb movement index; PSG: polysomnography

References:

1. Li Y, Lei F, Du L, Sun Y, Yang L, Tang X: Follow-up management of refractory continuous positive airway pressure therapy: emergent periodic limb movements in sleep. Sleep Med. 2014; 15:1165-7.
2. Li Y, Li Z, Lei F, Zhou J, Tang X: Daytime leg pain after overnight continuous positive airway pressure titration. J Neurol Sci. 2014; 336:281-3.
3. Chirakalwasan N, Hassan F, Kaplish N, Fetterolf J, Chervin RD: Near resolution of sleep related rhythmic movement disorder after CPAP for OSA. Sleep Med. 2009; 10:497-500.
4. Stoohs RA, Blum HC, Suh BY, Guilleminault C: Misinterpretation of sleep-breathing disorder by periodic limb movement disorder. Sleep Breath. 2001; 5:131-7.
5. McCall WV, Haponik E: Relationship of nasal continuous positive airway pressure to periodic limb movement disorder in a patient without sleep apnea. Chest. 1993; 103:1609-11.
6. Seo WH, Guilleminault C: Periodic leg movement, nasal CPAP, and expiratory muscles. Chest. 2012; 142:111-8.

7. Xie J, Covassin N, Chahal AA, Schulte PJ, Singh P, Somers VK, Caples SM: Effect of Adaptive Servo-Ventilation on Periodic Limb Movements in Sleep in Patients With Heart Failure. Am J Cardiol. 2019; 123:632-7.
8. Pai V, Khatwa U, Ramgopal S, Singh K, Fitzgerald R, Kothare SV: Prevalence of pediatric periodic leg movements of sleep after initiation of PAP therapy. Pediatr Pulmonol. 2014; 49:252-6.
9. Nikolopoulou M, Byraki A, Ahlberg J, Heymans MW, Hamburger HL, De Lange J, Lobbezoo F, Aarab G: Oral appliance therapy versus nasal continuous positive airway pressure in obstructive sleep apnoea syndrome: a randomised, placebo-controlled trial on self-reported symptoms of common sleep disorders and sleep-related problems. J Oral Rehabil. 2017; 44:452-60.
10. Schipper MH, Alvarez-Estevez D, Jellema K, Verbraecken J, Fulda S, Rijsman RM: Sleep-related leg movements in obstructive sleep apnea: definitions, determinants, and clinical consequences. Sleep Med. 2020; 75:131-40.
11. Singh B, Singh AP, Attri JP: Randomized Controlled Trial to Compare Baska(R) Mask versus ProSeal Laryngeal Mask Airway for General Anesthesia with Intermittent Positive Pressure Ventilation. Anesth Essays Res. 2020; 14:25-8.
12. Veeravigrom M, Desudchit T: Prevalence of Sleep Disorders in Thai Children. Indian J Pediatr. 2016; 83:1237-41.
13. Wu MN, Lai CL, Liu CK, Liou LM, Yen CW, Chen SC, Hsieh CF, Hsieh SW, Lin FC, Hsu CY: More severe hypoxemia is associated with better subjective sleep quality in obstructive sleep apnea. BMC Pulm Med. 2015; 15:117.
14. Charciunaite K, Gauronskaite R, Slekyte G, Danila E, Zablockis R: Evaluation of Obstructive Sleep Apnea Phenotypes Treatment Effectiveness. Medicina (Kaunas). 2021; 57.
15. Manconi M, Fanfulla F, Ferri R, Miano S, Haba-Rubio J, Heinzer R, Horvath T, Proserpio P, Young P, Moschovitis G, et al: Periodic limb movements during sleep in stroke/TIA: Prevalence, course, and cardiovascular burden. Neurology. 2018; 90:e1663-e72.
16. Guerrero ML, Kim D, Rupp TL, Balkin TJ: Oral appliance titration in patients with obstructive sleep apnea induces the appearance of periodic limb movements. Sleep Breath. 2010; 14:359-63.
17. Dongol EM, Williams AJ: Residual excessive sleepiness in patients with obstructive sleep apnea on treatment with continuous positive airway pressure. Curr Opin Pulm Med. 2016; 22:589-94.

eTable 4: Jadad scores of the included studies

Author (year)	Randomization	Blinding	Account of patient	Total score	Design	Country
Budhiraja, R. (2020)	1	1	1	3	Randomized controlled trial	USA
Murase, K. (2020)	0	0	1	1	Prospective cohort study	Japan
Wu, M.N. (2018)	0	0	0	0	Retrospective chart review	Taiwan
Yang, X.R. (2018)	0	0	0	0	Retrospective cohort study	China
Mwenge, G.B. (2017)	0	0	0	0	Retrospective cohort study	Belgium
Aritake-Okada, S. (2012)	0	0	1	1	Prospective cohort study	Japan
Hedli, L.C. (2012)	0	0	1	1	Prospective cohort study	USA
Benz, R.L. (2011)	0	0	1	1	Prospective cohort study	USA
Drigo, R. (2006)	0	0	1	1	Prospective cohort study	Italy
Baran, A.S. (2003)	0	0	1	1	Prospective cohort study	USA
Noseda, A. (2002)	0	0	1	1	Prospective cohort study	Belgium
Carelli, G. (1999)	0	0	1	1	Prospective cohort study	France
Yamashiro, Y. (1994)	0	0	1	1	Prospective cohort study	Canada
Fry, J.M. (1989)	0	0	1	1	Prospective cohort study	USA
