# Supplementary material for: Changes in Periodic Limb Movements of Sleep After the Use of Continuous Positive Airway Pressure Therapy: A Meta-Analysis
Source: Front Neurol. 2022 Jun 2;13:817009. doi: 10.3389/fneur.2022.817009 (PMC9202316; doi:10.3389/fneur.2022.817009)
Supplement: Supplementary file 2 [file Presentation_1.pdf]

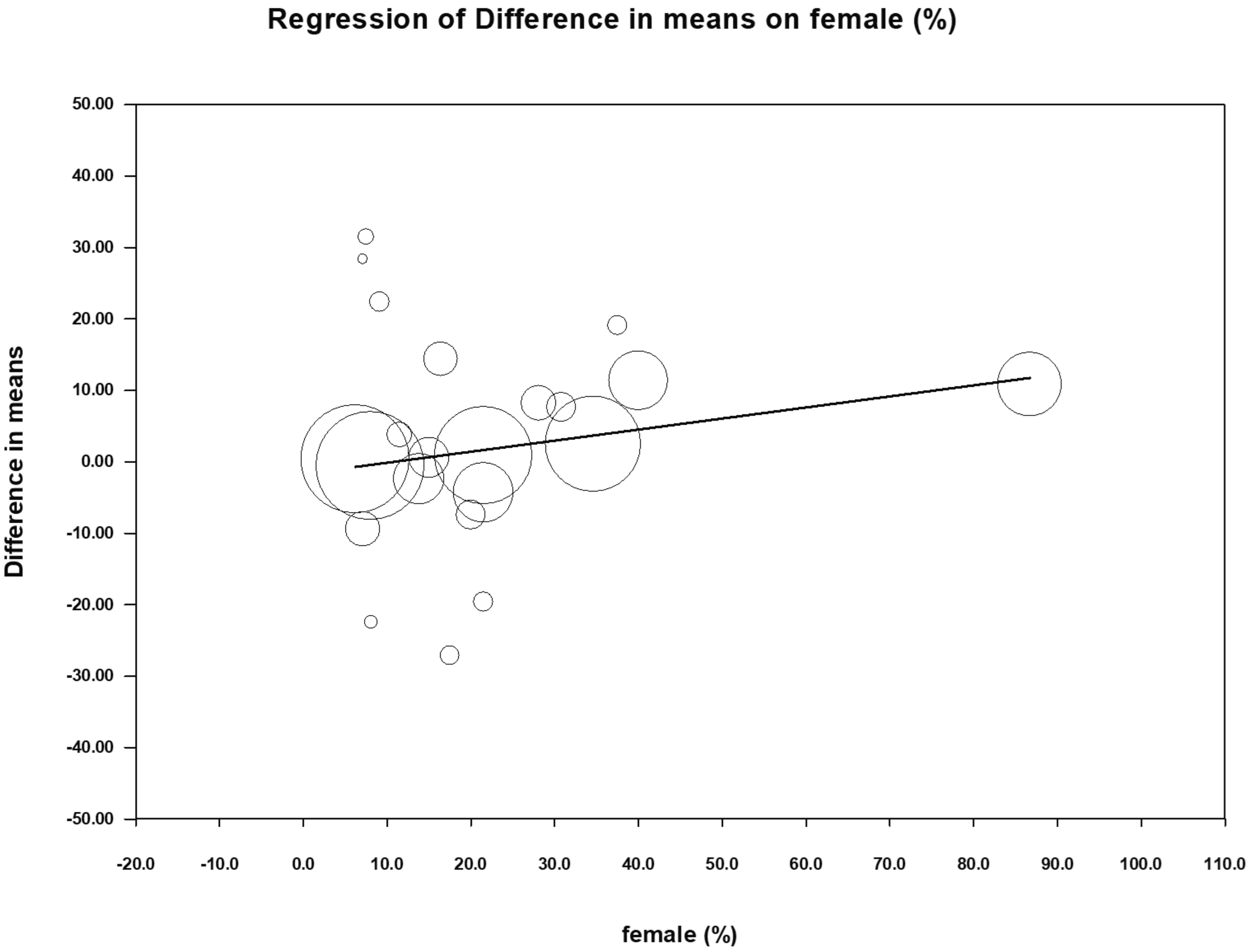

eFigure 1A meta-regression between changes of PLMI and female proportion

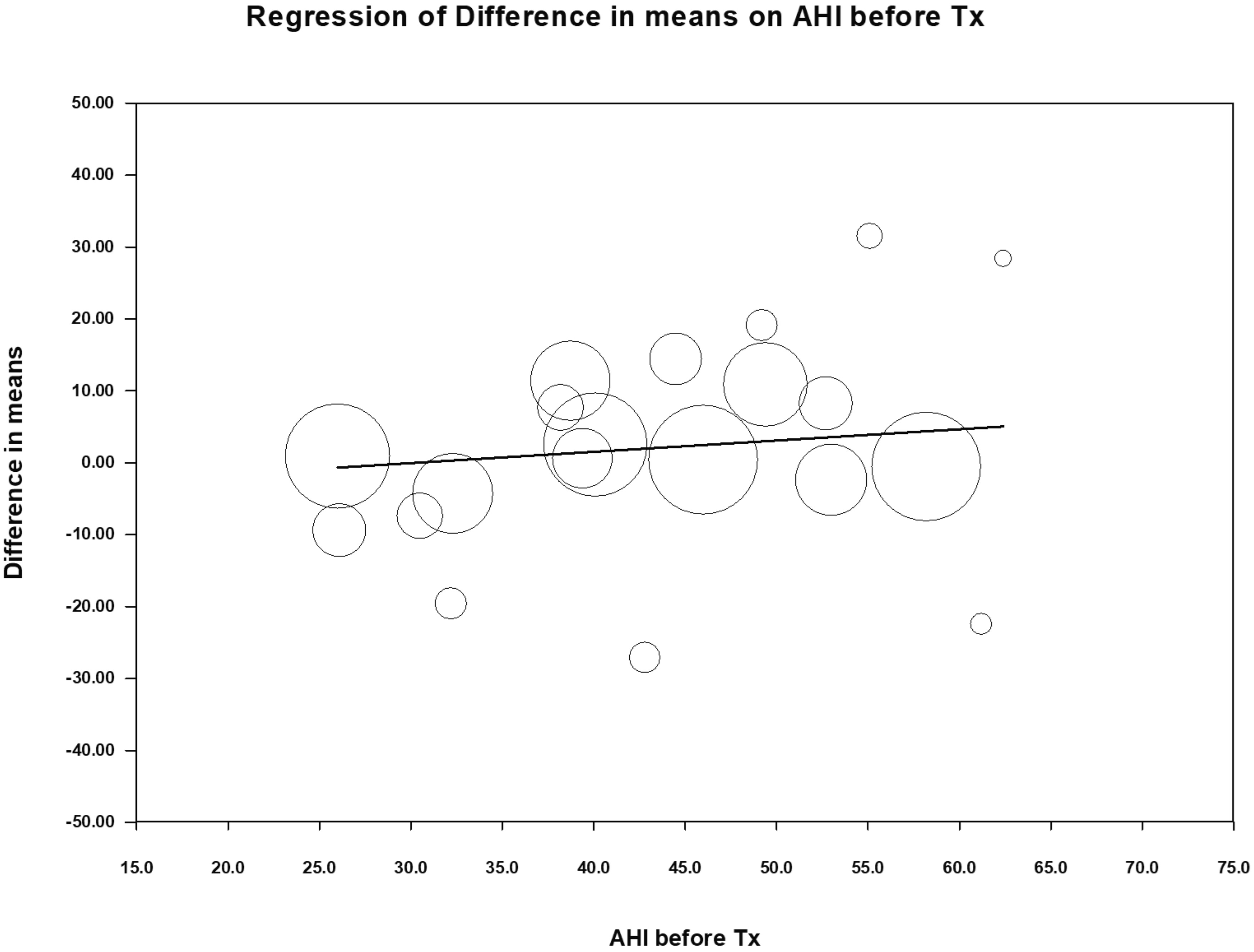

eFigure 1B meta-regression between changes of PLMI and baseline AHI

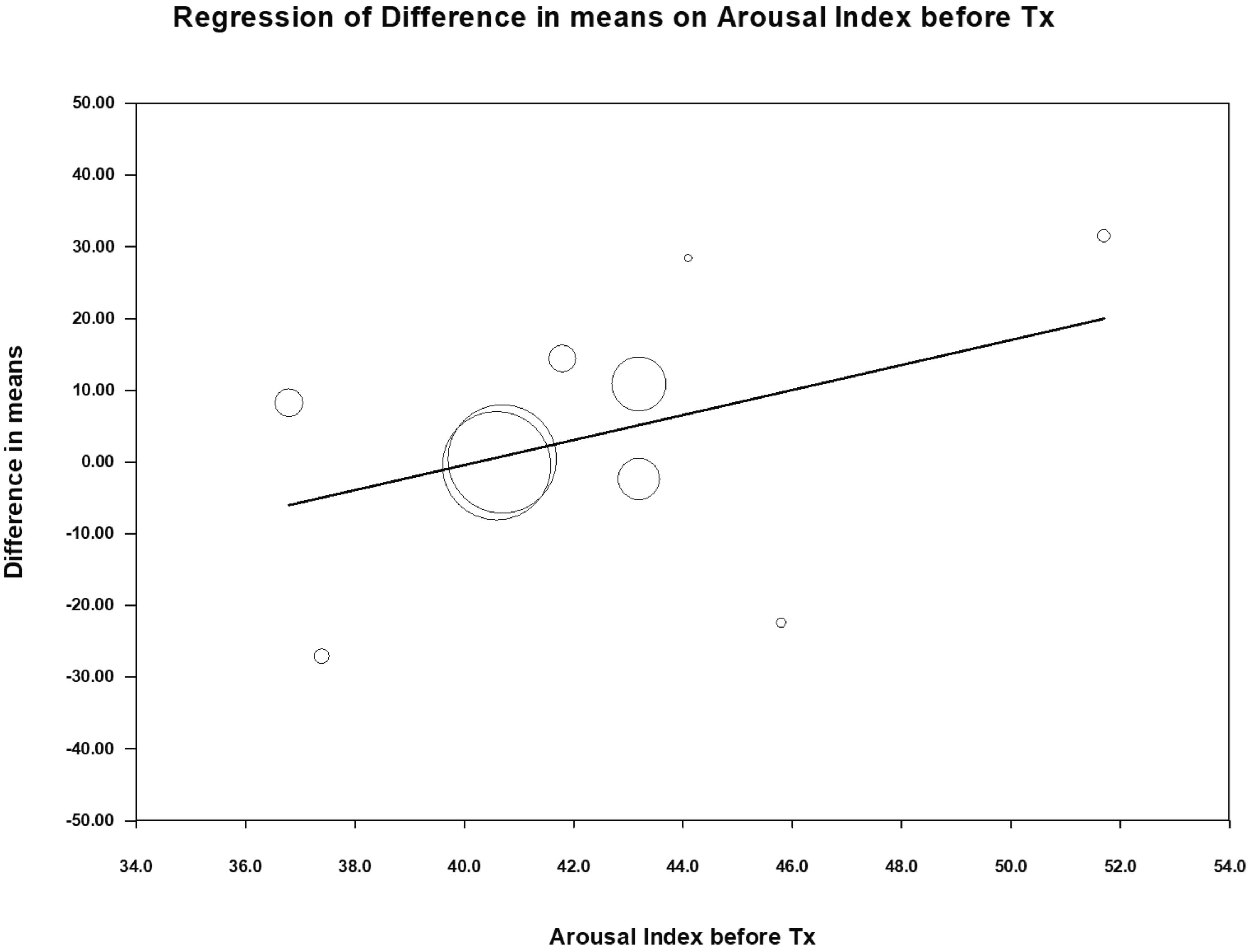

eFigure 1C meta-regression between changes of PLMI and baseline arousal index

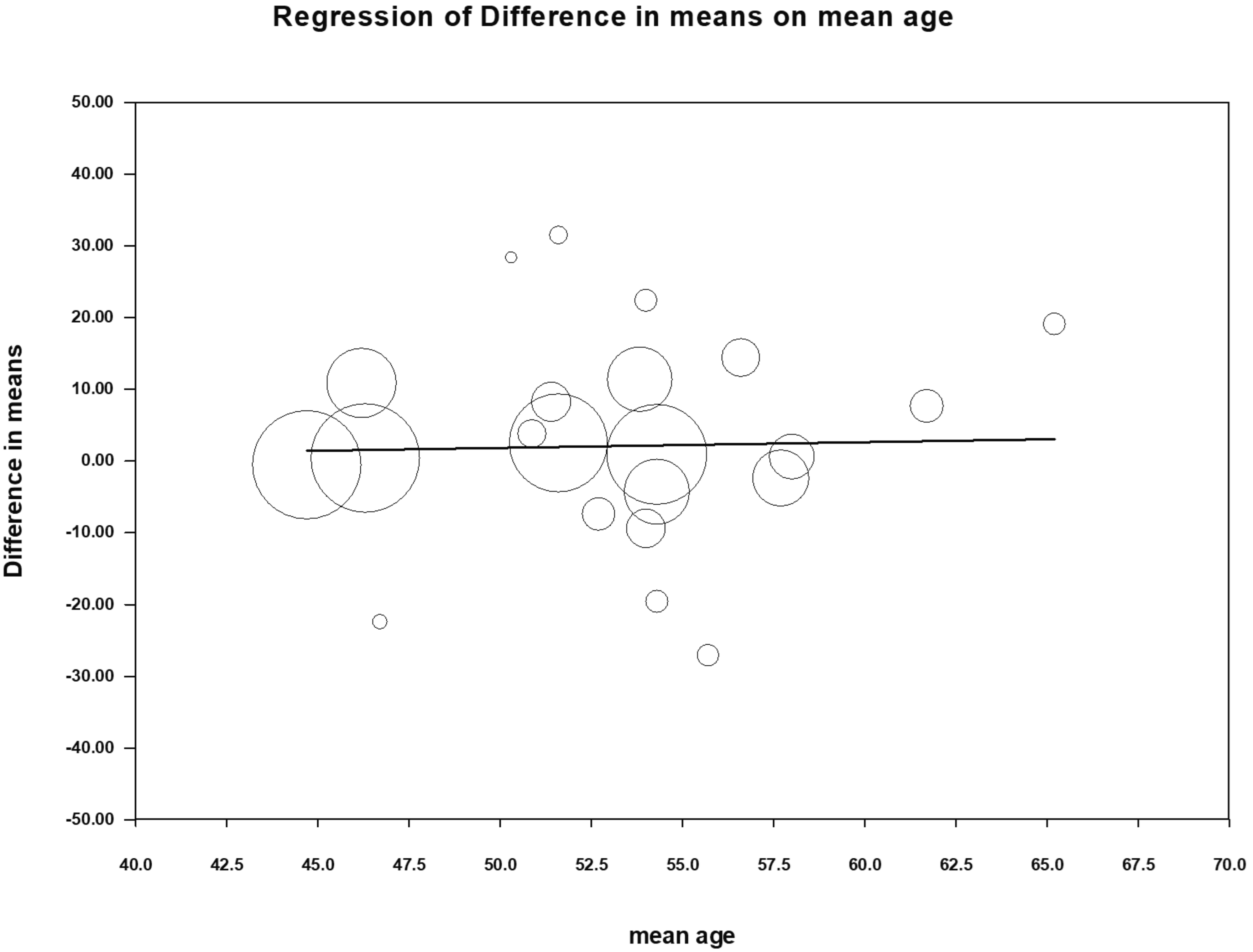

eFigure 1D meta-regression between changes of PLMI and mean age

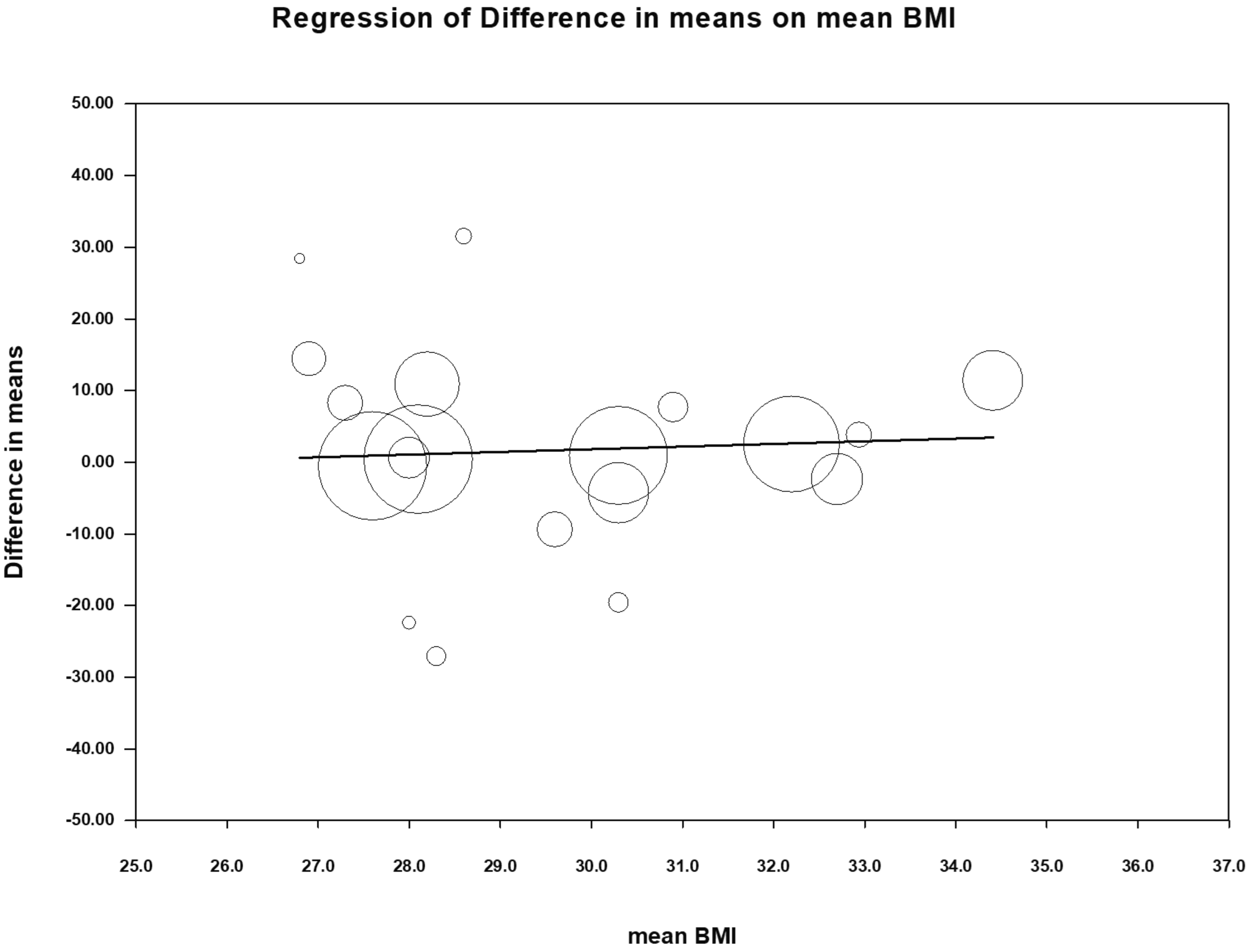

eFigure 1E meta-regression between changes of PLMI and mean BMI

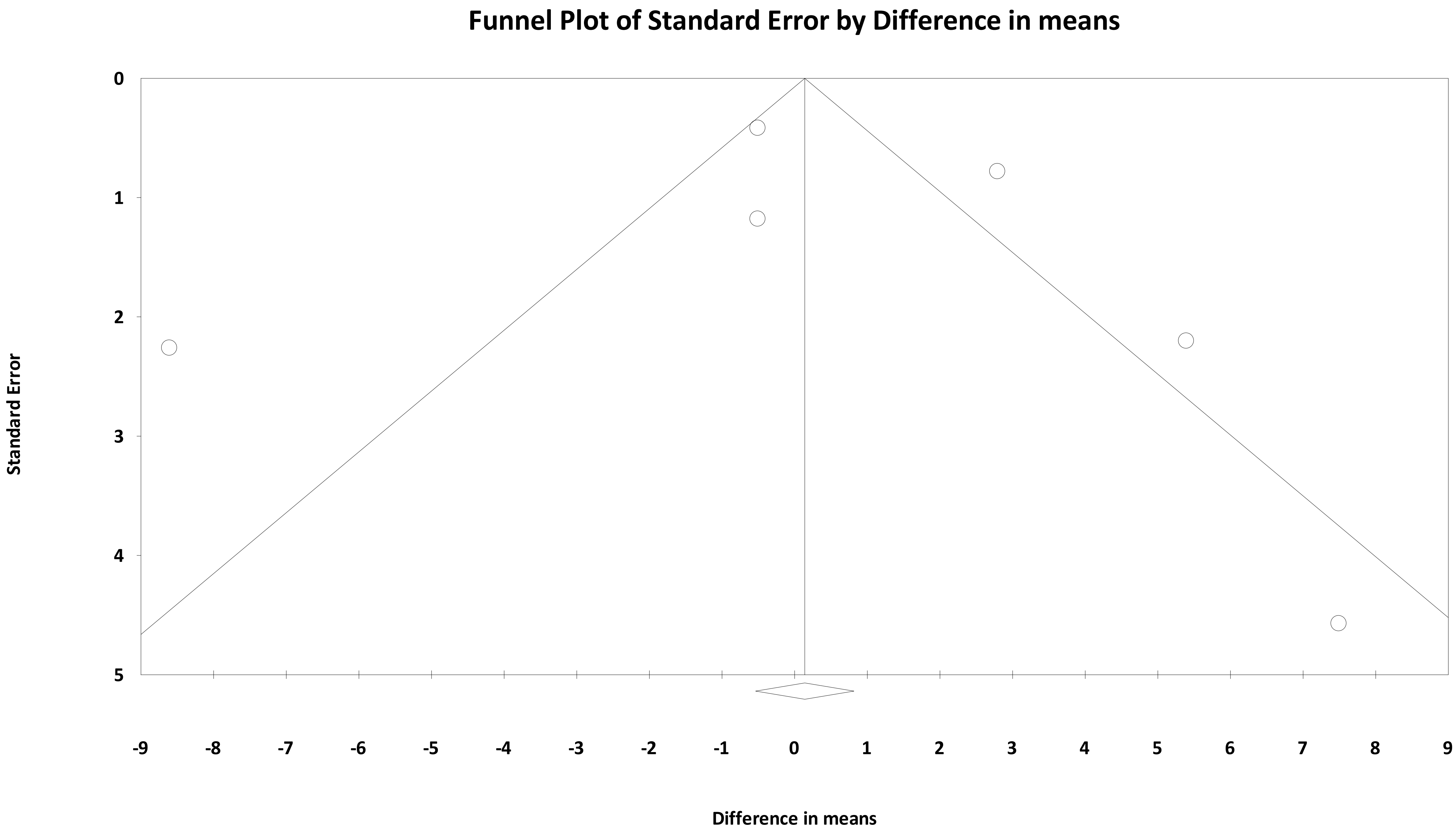

eFigure 2 Funnel plot of changes of PLMAI before and after CPAP administration
